# The Effects and Mechanisms Underlying Red Meat Diet on Endometriosis Through Modulating Gut Microbiota

**DOI:** 10.1002/fsn3.72277

**Published:** 2026-08-24

**Authors:** Mengru Li, Qian Peng, Yang Li, Qian Liu, Aili Tan, Jinxin Xiao, Wenwei Chen, Jinhua Tian, Suting Li, Qingzhen Xie

**Affiliations:** ^1^ Department of Obstetrics and Gynecology, Renmin Hospital Wuhan University Wuhan China; ^2^ Department of Pathology, Zhongnan Hospital Wuhan University Wuhan China; ^3^ Centre for Reproductive Medicine, Renmin Hospital Wuhan University Wuhan China

**Keywords:** endometriosis, gut microbiome, red meat diet, TLR4/NFκB signaling pathway

## Abstract

Red meat consumption is widespread globally and has been associated with endometriosis (EMS) progression, although the precise underlying mechanisms remain unclear. In the present study, we aimed to explore the mechanisms by which a red meat diet exacerbates endometriosis progression. Our results showed that red meat intake significantly promoted the growth of endometriotic lesions and aggravated the related inflammatory responses. Mechanistically, a red meat diet alters the composition of the gut microbiota, decreases the production of short‐chain fatty acids (SCFAs), impairs intestinal barrier function, and activates systemic inflammatory signaling pathways. These findings illustrate that red meat consumption accelerates endometriosis progression through a gut microbiota‐mediated pathway, providing new insights into dietary intervention strategies for endometriosis.

## Graphical abstract

A red meat diet induces gut microbiota dysbiosis, characterized by the expansion of Enterobacteriaceae and *Enterococcus* and depletion of SCFA‐producing bacteria, leading to reduced intestinal SCFA levels. This impairs intestinal barrier integrity, promotes LPS leakage into the circulation and peritoneal cavity, and activates the TLR4/MYD88/NF‐κB signaling pathway, ultimately exacerbating endometriosis progression. SCFA supplementation partially reversed these effects, suggesting a potential therapeutic strategy for endometriosis via gut microbiota modulation.

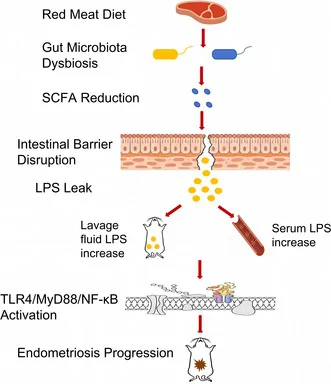

**Abbreviations**
AABX_FMT (CD + EMS): the FMT (CD + EMS) group after antibiotic treatment
AABX_FMT (RMD + EMS): the FMT (RMD + EMS) group after antibiotic treatment
BABX_FMT (CD + EMS): the FMT (CD + EMS) group before antibiotic treatment
BABX_FMT (RMD + EMS): the FMT (RMD + EMS) group before antibiotic treatment
CD: control diet
ELISA: enzyme linked immunosorbent assay
EMS: endometriosis
FMT: fecal microbiota transplantation
GC–MS: gas chromatography–mass spectrometry
HE: hematoxylin–eosin
IL: interleukin
LPS: lipopolysaccharide
MYD88: myeloid differential protein 88
NF‐κB: nuclear factor‐kappa B
P65: protein 65
PCoA: principal coordinate analysis
PP65: phosphoprotein
RMD: red meat diet
SCFA: short chain fatty acid
SPF: specific pathogen‐free
TLR4: toll‐like receptor 4
TNF‐α: tumor necrosis factor α
WB: western blot
ZO‐1: zonula occludens

## 1 Introduction

Endometriosis is a long‐term inflammation‐related disorder identified by endometrial‐similar lesions outside the uterus (International Working Group of AAGL et al. 2021). Worldwide, roughly 10% of women of childbearing age are affected by this disease (Ghiasi et al. 2020). The predominant medical manifestations of endometriosis are pelvic ache and sterility, which have serious negative impacts on the well‐being of affected women (Koller et al. 2023). The pathogenesis of endometriosis is multifaceted and remains incompletely understood.

Dietary patterns are closely associated with the risk of and progression of endometriosis (Dougan et al. 2024; Oszajca and Adamus 2024). Notably, red meat exhibits pro‐inflammatory characteristics and is significantly associated with an increased risk of endometriosis (Yamamoto et al. 2018; Ley et al. 2014; Wu, Shinde, et al. 2021; Wu, Zhang, et al. 2021; Shiraseb et al. 2022; Samraj et al. 2015). Dietary interventions targeting intestinal inflammation have shown potential for relieving endometriosis‐related pain and improving quality of life (Piecuch et al. 2022; van Haaps et al. 2023). However, the specific mechanisms by which red meat intake affects endometriosis remain unclear.

The intestinal microbiota consists mainly of bacteria, archaea, and fungi (Simpson et al. 2023), which colonize the gastrointestinal tract and regulate various physiological functions. Gut microbiota is critical for maintaining intestinal homeostasis, immune system development, metabolic homeostasis, and immune and inflammatory responses (Schluter et al. 2020; Zheng et al. 2020; Trompette et al. 2014; Roy and Trinchieri 2017; Belkaid and Hand 2014; Ye et al. 2026; Huang et al. 2025). Dietary patterns can significantly reshape the gut microbiota composition and function, which in turn modulates metabolism and systemic inflammation (Yang and Cong 2021; Wastyk et al. 2021). Multiple studies have shown that a diet high in red meat can alter the composition of the gut microbiota, thereby affecting health (Larsson et al. 2025; Liu et al. 2023).

Growing evidence supports a close link between gut microbiota dysbiosis and endometriosis pathophysiology. Disturbances in gut microbiota may contribute to disease progression (Chadchan et al. 2023; Parpex et al. 2024; Yuan et al. 2018). In addition to retrograde menstruation, chronic inflammation and immune dysfunction play key roles in endometriosis. Immune cell abnormalities and excessive pro‐inflammatory mediators drive local and systemic inflammation, which is central to the onset and development of endometriosis (Dai et al. 2025; Blanco et al. 2025). TLR4/NF‐κB is a classic inflammatory signaling pathway that plays a significant role in immune regulation and inflammatory responses, and is involved in the pathological processes of endometriosis are closely related to endometriosis (Su et al. 2021; Yun et al. 2016). Furthermore, numerous studies have shown that alterations in the gut microbiota and damage to the intestinal barrier are involved in the activation of the TLR4/NF‐κB pathway, thereby promoting the progression of the disease (Du et al. 2025; Meng et al. 2025; Liu et al. 2024).

However, the mechanism by which a red meat diet regulates the gut microbiota and the downstream TLR4‐mediated inflammatory pathway to affect endometriosis remains poorly defined. Therefore, the present study aimed to explore whether a red meat diet promotes the development of endometriotic lesions and to clarify the underlying mechanisms involving the gut microbiota and related inflammatory signaling.

## 2 Materials and Methods

### 2.1 Animal Experiments

Eight‐week‐old female C57BL/6‐N mice (weight, 20–22 g) were obtained from the Animal Experiment Center of Renmin Hospital of Wuhan University to establish endometriosis models. All animal experimental procedures in this research were authorized by the Animal Ethics Committee of Renmin Hospital, Wuhan University (IACUC issue no. WDRM20240505C). All experimental processes were strictly in conformity with the national standards of China, namely, the “Guidelines for the Care and Use of Laboratory Animals” (GB/T 35892–2018) and “Guidelines for the Ethical Review of Laboratory Animal Experiments” (GB/T 35823–2018), and followed the international ARRIVE reporting guidelines to maximize animal welfare. The mice in our study were housed under a standard 12‐h light/12‐h dark cycle in a specific pathogen‐free (SPF) environment with unlimited access to food and water. Before the experiment, the mice underwent a 10‐day acclimatization period, during which daily vaginal smears were obtained to monitor their estrous cycles. Mice that displayed at least two consecutive normal estrous cycles were selected for subsequent experimental.

**FIGURE 1:**
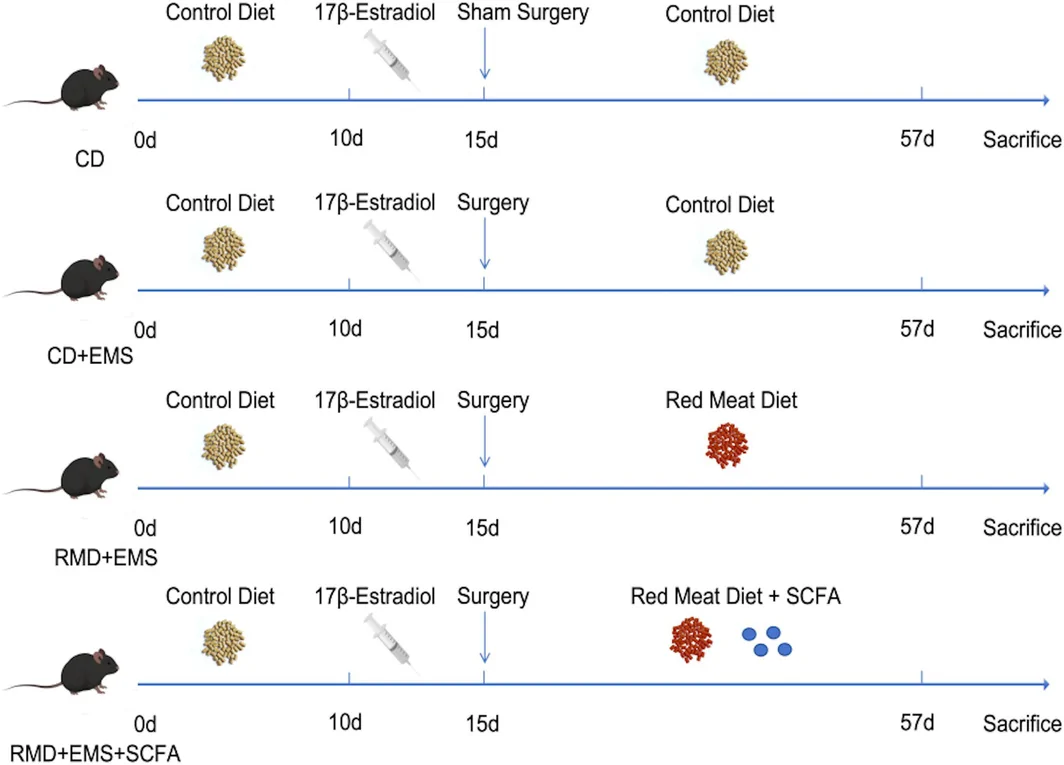
Part One: experimental design.

Before implantation, preoperative gavage was delivered to harmonize the reproductive phase of all mice, with each given 56 μg/kg/day of 17β‐estradiol for 5 days (Ingberg et al. 2012). Mice that underwent estrous cycle screening and synchronization were randomly allocated to one of the groups (*n* = 5) (Figure 1): (1) CD: Control Diet, (2) CD + EMS: Control Diet + Endometriosis, (3) RMD + EMS: Red Meat Diet + Endometriosis, (4) RMD + EMS + SCFA: Red Meat Diet + Endometriosis + Short Chain Fatty Acid.

In this study, the CD group underwent sham surgery, whereas the other three groups were subjected to auto transplantation to induce endometriosis. The specific method for constructing the mouse endometriosis model was as follows: After administering isoflurane anesthesia, tracheal intubation and mechanical ventilation were performed on the mice. A midline incision was made on the ventral side, and one side of the uterine horn was removed. An endometrial tissue block of approximately 3 mm was cut longitudinally and separated into sections. The tissue block was sutured and fixed to the peritoneal wall of the same mouse. In the sham operation group, only the suture was sewn onto the peritoneal wall without transplanting the endometrial tissue (Chadchan et al. 2019). The dosage of SCFAs was as follows: oral administration of sodium acetate (1.0 mg/g, once daily) (He et al. 2024). The ratio of the red meat diet was based on the low‐dose red meat diet ratio from previous studies (Li et al. 2021). To ensure the consistency of cornstarch content in all groups, the cornstarch content in all feeds was adjusted to 397.486 g/kg to eliminate the potential impact of resistant starch on SCFA synthesis (Sobh et al. 2022).

**FIGURE 2:**
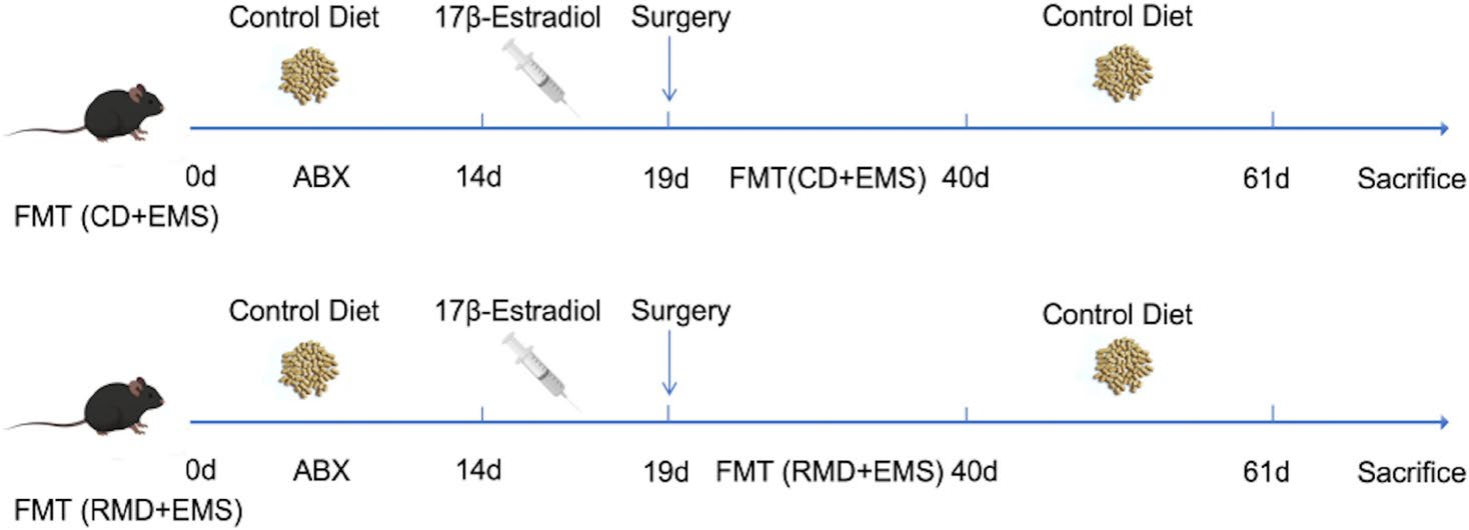
Part Two: experimental design.

To determine the causal association between intestinal flora and endometriosis onset, a fecal microbiota transplantation (FMT) experiment was performed. Feces from the CD + EMS and RMD + EMS groups were collected and transplanted into pseudo‐germfree mice to establish the two groups of models. The method of constructing pseudo‐germfree mice and FMT was the same as in previous studies (Geng et al. 2025). The groups were as follows (*n* = 5) (Figure 2): (5) FMT (CD + EMS): Control Diet + Endometriosis + FMT (CD + EMS); (6) FMT (RMD + EMS): Control Diet + Endometriosis + FMT (RMD + EMS).

### 2.2 Sample Collection and Processing

Mice were sacrificed 42 days after transplantation, and endometriotic lesions were surgically removed, counted, and weighed. The fixed tissues were encased in wax and sectioned at 5 μm. The sections were then subjected to hematoxylin and eosin staining and Ki67 immunohistochemistry. The sections were analyzed and imaged using a microscope at 200× magnification. Two independent observers manually quantified the proportion of Ki67‐positive cells, which were subjected to statistical analysis.

### 2.3 Fecal Microbiome Analysis

Fecal genomic DNA was extracted and amplified. The libraries were sequenced using the NovaSeq6000 platform. Effective sequences were obtained after quality filtering, assembly, and de‐chimerization of the raw data. Quantitative Insights Into Microbial Ecology 2 was utilized for species annotation and diversity analyses.α‐diversity was evaluated utilizing the Ace, Chao1, Shannon, and Simpson indices. β‐diversity was assessed utilizing principal coordinate analysis (PCoA) grounded on the Bray–Curtis distance. Disparities in species composition between groups were assessed using the Kruskal–Wallis test. The correlation between SCFAs levels and microbiome composition was evaluated using Spearman's correlation analysis.

### 2.4 SCFAs Analysis

The SCFAs content in feces was quantified by gas chromatography–mass spectrometry (GC–MS). Fecal specimens (20 mg) were extracted using a phosphoric acid solution, vortexed, sonicated, and centrifuged. The supernatant was blended with methyl tert‐butyl ether containing internal standards, and the organic phase was obtained after centrifugation for GC–MS/MS analysis. A Metware Database was performed based on the standards for qualitative analysis of mass spectrometry data. Quantification was performed using the multiple reaction monitoring mode of a triple quadrupole mass spectrometer.

### 2.5 ELISA and WB Analysis

The levels of lipopolysaccharide (LPS), interleukin‐1β (IL‐1β), interleukin‐6 (IL‐6), and tumor necrosis factor α (TNF‐α) in mouse peritoneal lavage fluid and serum were quantified using an ELISA kit (ABclonal Biotechnology Co. Ltd.) as described previously (Ni et al. 2021). Total proteins were extracted from mouse colon and endometriotic lesion tissues after liquid nitrogen grinding, and western blotting (WB) was performed as previously explained (Dong et al. 2022). The antibodies used included zonula occludens (ZO‐1) (Cell Signaling Technology), Claudin 1, Occludin, toll‐like receptor 4 (TLR4), myeloid differential protein 88 (MYD88), phosphoprotein (PP65), and protein 65 (P65) (Affinity Biosciences). The anti‐phospho‐P65 antibody used in this study specifically targeted the Ser536 phosphorylation site of P65. The bands were exposed with e‐blot, and quantitative analysis was performed using Image J (Java 1.8.0_172) and GraphPad Prism (version 10.3.1).

## 3 Result

### 3.1 Red Meat Diet Aggravates Endometriosis in Mice

**FIGURE 3:**
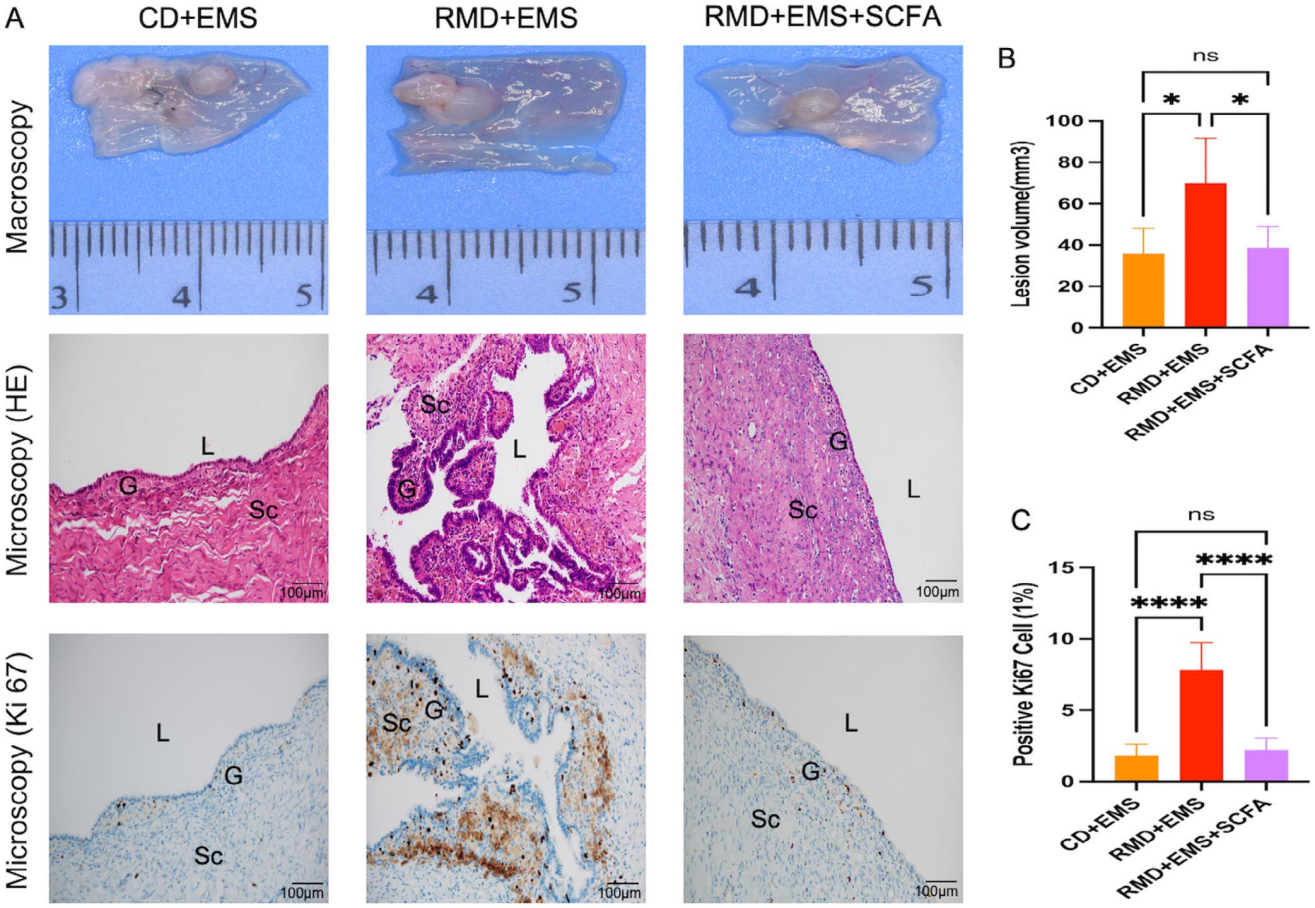
Development of endometriosis lesions following different diets. (A) Macroscopic observation images, HE staining images, and Ki67 immunohistochemical images of the implants in the CD + EMS, RMD + EMS, and RMD + EMS + SCFA groups on Day 57. (B) Lesion volumes of the CD + EMS, RMD + EMS, and RMD + EMS + SCFA groups on Day 57. (C) Percentage of epithelial cells with positive Ki‐67 immunostaining in the CD + EMS, RMD + EMS, and RMD + EMS + SCFA groups (G, glandular cells; L, lumen; Sc, stromal cells). One‐way ANOVA. ns, non‐significant; *p < 0.05, **p < 0.01, ***p < 0.001. Statistics are shown as mean ± SE (*n* = 5).

Compared with the CD + EMS and RMD + EMS + SCFA groups, mice in the RMD + EMS group had larger endometriosis lesions. However, there was no substantial variation in the size of endometriotic lesions between the CD + EMS and RMD + EMS + SCFA groups (Figure 3A,B).

HE staining showed that the mice used to construct the endometriosis model exhibited characteristic endometriosis‐like structures (epithelial layer and glandular area). However, compared with the CD + EMS and RMD + EMS + SCFA groups, the lesions in the RMD + EMS group had a thicker epithelial layer and more glands (Figure 3A). Ki‐67 immunohistochemical staining was performed to detect cell proliferation (Figure 3A). The observations demonstrated that the proliferation index of the lesions in the RMD + EMS group was markedly higher than that observed in the CD + EMS and RMD + EMS + SCFA groups (*p* < 0.05) (Figure 3C).

### 3.2 Red Meat Diet Aggravates Intestinal Microbiota Disturbance in Mice With Endometriosis

Recognizing that a diet high in red meat is pro‐inflammatory, it affects the constitution of the gut flora (Li et al. 2021; Zhang et al. 2019). Thus, we used 16S rRNA gene sequencing to identify the taxonomic characteristics of the gut microflora in each mouse group.

**FIGURE 4:**
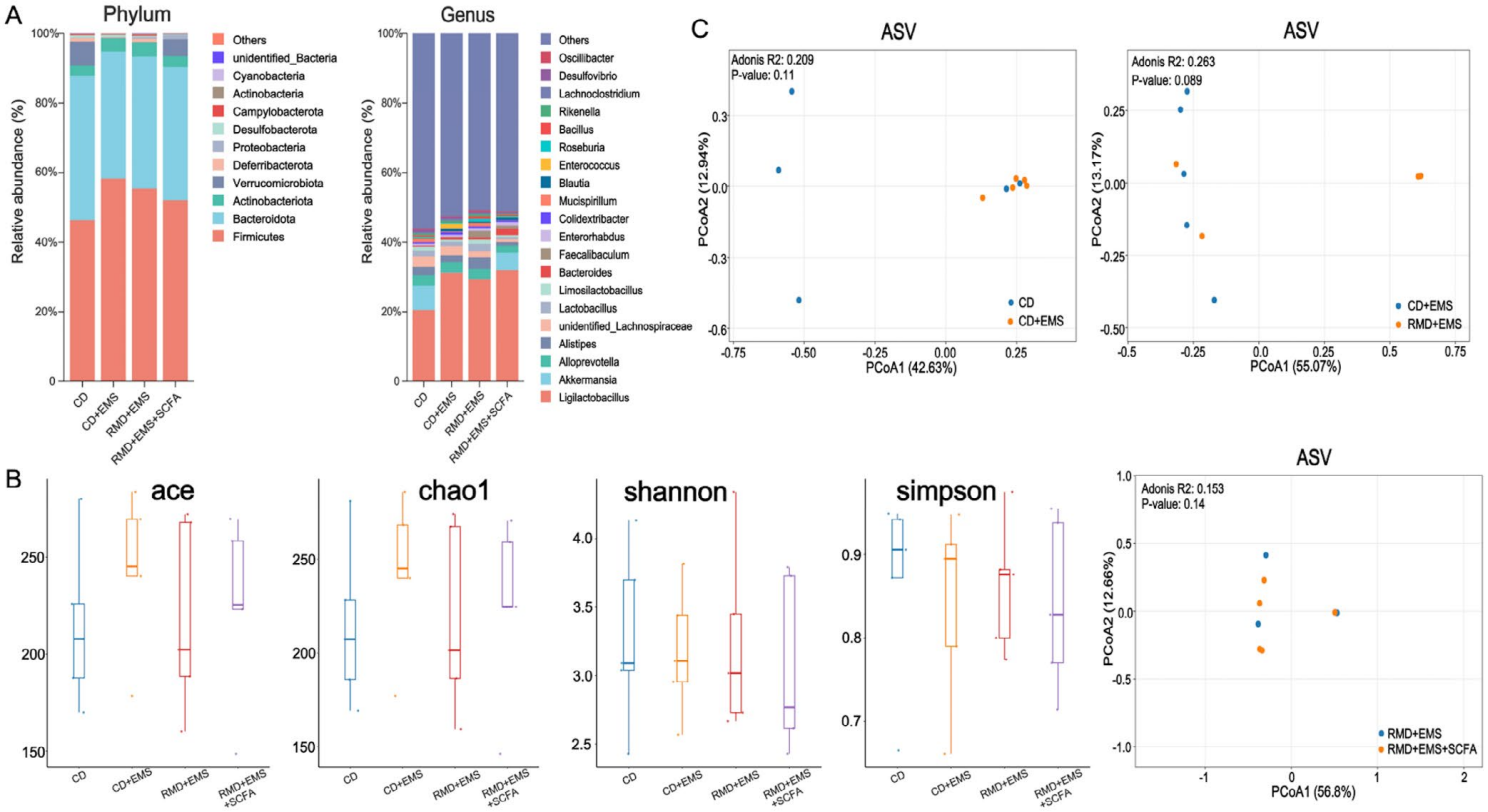
Gut microbiota composition of the CD, CD + EMS, RMD + EMS, and RMD + EMS + SCFA groups on Day 0. (A) Phylum‐ and genus‐level constitution of the gut flora in the four groups. (B) Alpha diversity index of the intestinal microbiota within each group. (C) Beta diversity of bacteria based on PCoA according to the Bray–Curtis distance between the four groups at the genus rank. *p < 0.05, **p < 0.01 (Kruskal–Wallis test or one‐way ANOVA) (*n* = 5).

Before the experiment, we detected the gut microbiome of each group of mice. The five most prevalent species were Firmicutes, Bacteroidota, Actinobacteriota, Verrucomicrobiota, and Deferribacterota at the phylum level (Figure 4A). Each group of mice had a rich microbiota containing genera such as *Ligilactobacillus*, *Akkermansia*, *Alloprevotella*, *Alistipes*, *unidentified_Lachnospiraceae*, and *Lactobacillus* at the genus level (Figure 4A). The alpha diversity index of the gut microflora of each group of mice was calculated based on the ASV levels. The Ace, Chao1, Shannon, and Simpson indices indicated no statistically significant variation (Figure 4B). The PCoA diagram of the Bray–Curtis distance showed that at the beginning of the experiment, there was no significant separation of the gut flora among the different groups of mice (Figure 4C).

**FIGURE 5:**
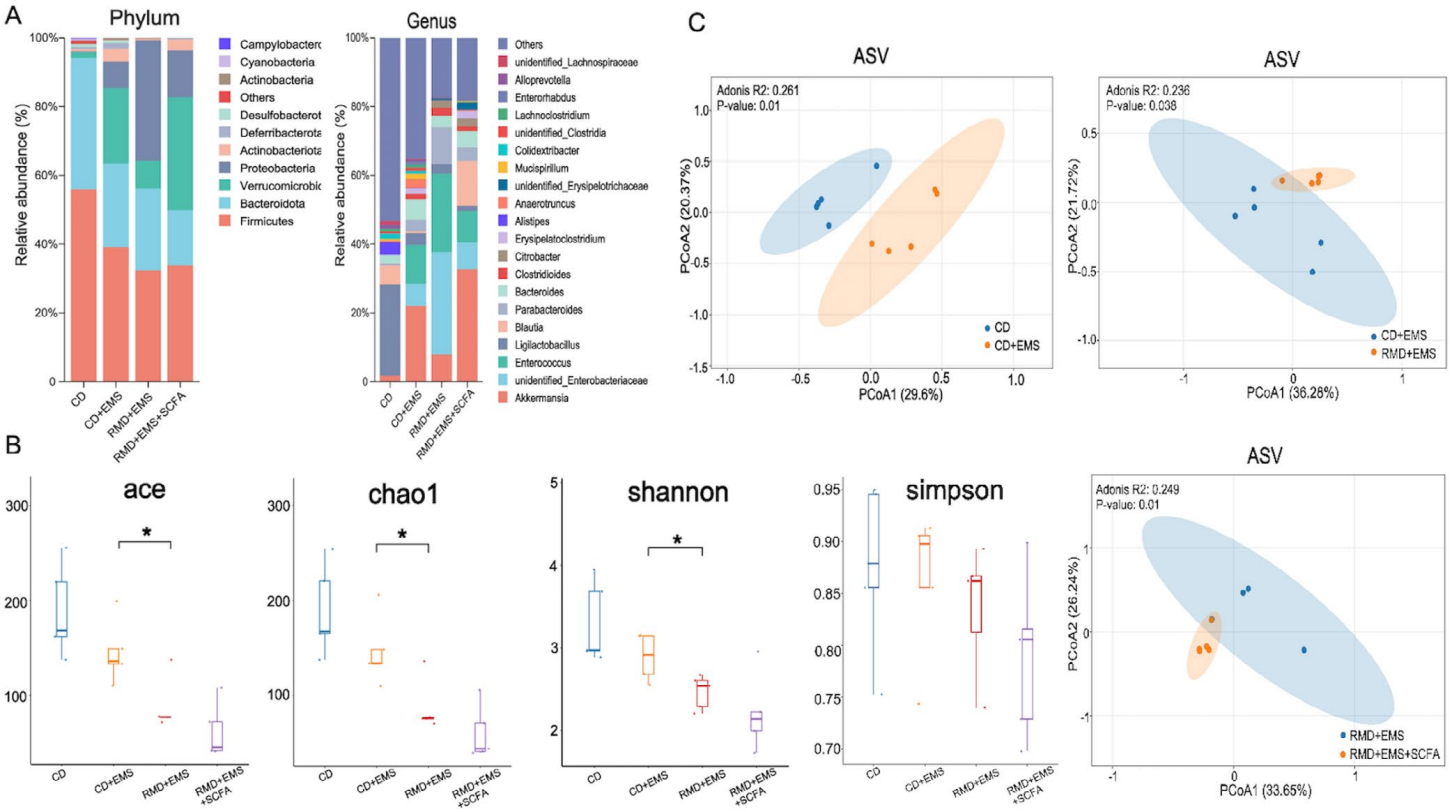
On Day 57, gut microbiota of the CD, CD + EMS, RMD + EMS, and RMD + EMS + SCFA groups. (A) Phylum‐ and genus‐level constitution of the gut flora in the four groups. (B) Alpha diversity index of the intestinal microbiota within each group. (C) Beta diversity of bacteria based on PCoA according to the Bray–Curtis distance between the four groups at the genus rank. *p < 0.05, **p < 0.01 (Kruskal–Wallis test or one‐way ANOVA) (*n* = 5).

After concluding the experiment, we analyzed the gut microflora of each group of mice. The five most prevalent species were Firmicutes, Bacteroidota, Verrucomicrobiota, Proteobacteria, and Actinobacteriota at the phylum level (Figure 5A). The main microflora differed among the four groups at the genus level (Figure 5A). Compared with the normal control group (CD) mice, CD + EMS mice exhibited intestinal microbiota dysbiosis, distinguished by decreased prevalence of *Ligilactobacillus* and *Blautia*, whereas the prevalence of *Akkermansia*, *Enterococcus*, *unidentified‐Enterobacteriaceae*, *Parabacteroides*, and *Bacteroides* increased (Figure 5A). Notably, compared with the CD + EMS group, red meat diet feeding elevated the quantities of *Enterococcus*, *unidentified‐Enterobacteriaceae*, and *Parabacteroides* and diminished the quantities of *Lactilactobacillus*, *Blautia*, and *Akkermansia* (Figure 5A), but SCFAs intervention reversed the alterations in the intestinal microbiota induced by the red meat diet.

The alpha diversity index of the intestinal microflora in each group of mice was calculated based on ASV levels (Figure 5B). Although there was no statistical significance in the Ace, Chao1, and Shannon indices of the intestinal microbiota of CD + EMS group mice compared to that of CD group mice, the overall trend was a decrease, demonstrating that the species abundance in the intestinal microflora might have decreased. The Ace, Chao1, and Shannon indices of the gut microflora of the RMD + EMS group mice were substantially lower than those of the CD + EMS group mice (*p* < 0.05), suggesting that the red meat diet reduced the species richness of the microbial community. However, there was no statistical significance in the comparison of the Ace, Chao1, Shannon, and Simpson indices of the intestinal microflora between the RMD + EMS + SCFA group and the RMD + EMS group mice, although the overall trend was a decrease.

PCoA of the Bray–Curtis distance revealed that the initiation of endometriosis affected the structure of the gut microbiome (*R* = 0.261, *p* = 0.01) (Figure 5C). Dietary changes significantly altered the structure of the microflora between the CD + EMS and RMD + EMS groups and were independent of the presence of endometriosis (*R* = 0.236, *p* = 0.038) (Figure 5C). However, supplementing SCFAs in endometriosis mice fed a red meat diet could change the composition of the intestinal microbiota (*R* = 0.249, *p* = 0.01) (Figure 5C).

### 3.3 Red Meat Diet Inhibits the Concentration of SCFAs in the Intestines of Endometriosis Mice

**FIGURE 6:**
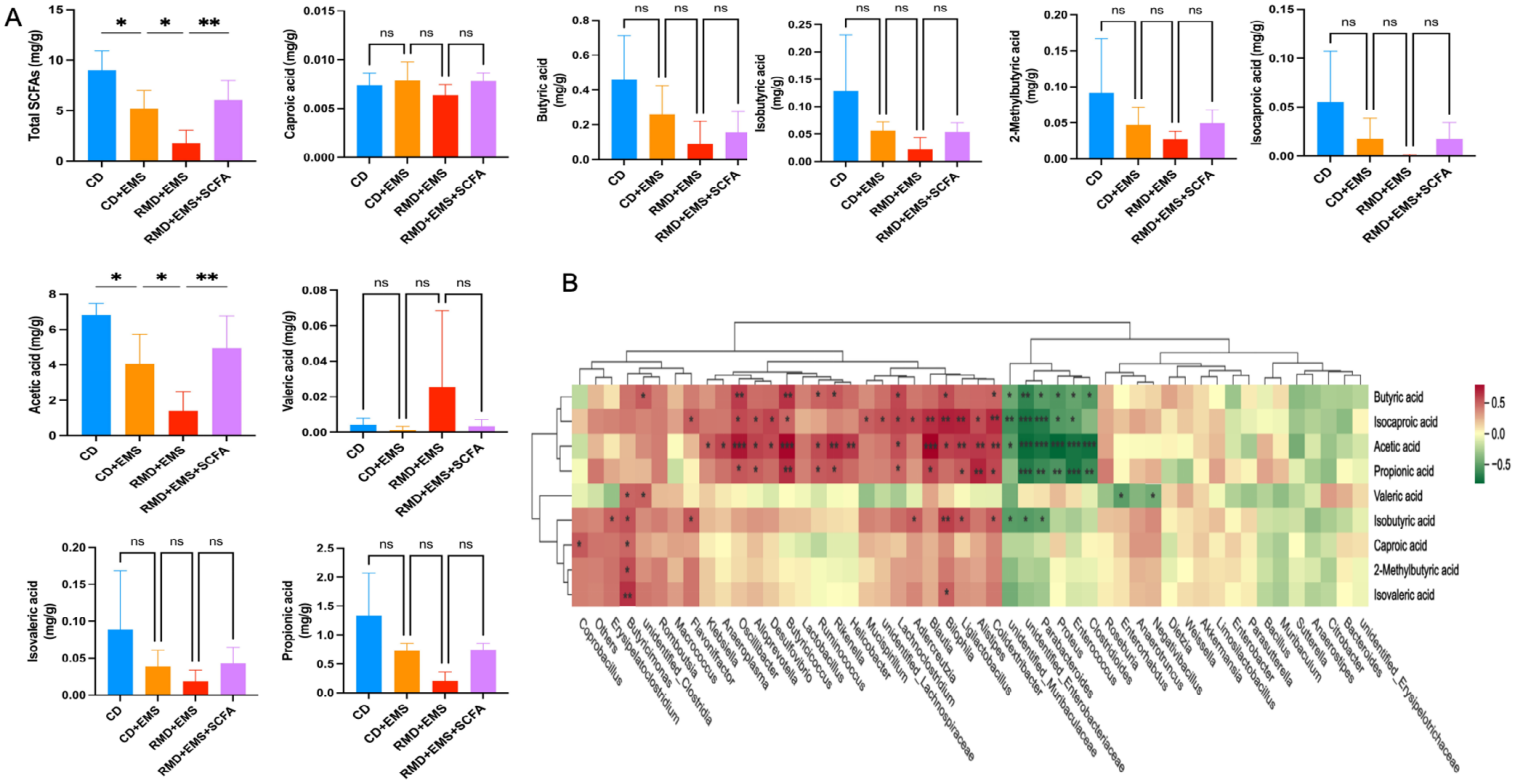
(A) On Day 57, total content of SCFAs and acetic acid in the CD, CD + EMS, RMD + EMS, and RMD + EMS + SCFA groups. One‐way ANOVA. ns, non‐significant; *p < 0.05, **p < 0.01. Statistics are presented as mean ± SE. (B) On Day 57, relationship between intestinal flora and SCFA concentrations. Spearman correlation analysis was executed between the top 50 most prevalent microbiota and SCFAs among the CD, CD + EMS, RMD + EMS, and RMD + EMS + SCFA groups. Green and red colors represent negative and positive relationships, respectively. *p < 0.05, **p < 0.01, ***p < 0.001 (*n* = 5).

SCFAs in the intestinal tract can affect gut barrier function and regulate the inflammatory response (Liu et al. 2021; Mann et al. 2024). As demonstrated in Figure 6A, the total concentrations of SCFAs in the intestines of mice with endometriosis were substantially reduced, and the intervention with a red meat diet further decreased the production of SCFAs in the intestines, mainly manifested as a decrease in acetic acid (Figure 6A). However, the intervention with SCFAs increased the SCFA content in the intestines of mice with endometriosis fed a red meat diet (Figure 6A). Through correlation analysis, it was found that the prevalence of flora such as *Blautia*, *Butyricicoccus*, *Oscillibacter*, and *Lachnoclostridium* were significantly positively linked to the concentrations of acetic acid and propionic acid; the prevalence of microbiota such as *Ligilactobacillus*, *Alloprevotella*, *Alistipes*, and *Ruminococcus* were significantly linked to the content of acetic acid; the quantities of *unidentified_Enterobacteriaceae*, *Parabacteroides*, *Proteus*, *Enterococcus*, and *Clostridioides* were inversely associated with the concentrations of acetic acid, propionic acid, and butyric acid (Figure 6B).

### 3.4 Red Meat Diet Compromises the Integrity of Gut Barrier and Exacerbates Inflammatory Response in Endometriosis Mice

**FIGURE 7:**
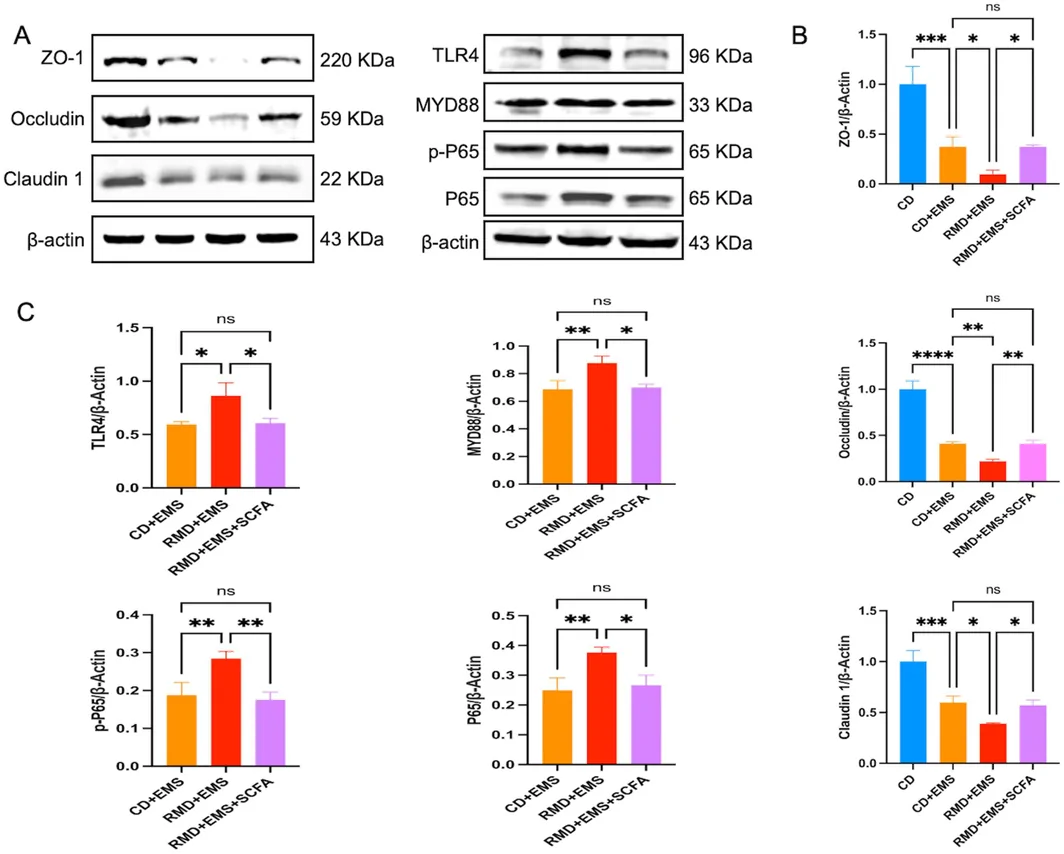
(A) Western blot results of intestinal barrier proteins (Zo1/Occludin/Claudin1) and the TLR4/MYD88/NF‐κB pathway in the CD, CD + EMS, RMD + EMS, and RMD + EMS + SCFA groups. (B) Relative amounts of ZO‐1, Occludin and Claudin1. (C) Relative amounts of TLR4, MYD88, p‐P65, and P65. One‐way ANOVA. ns, non‐significant; *p < 0.05, **p < 0.01, ***p < 0.001, ****p < 0.0001 (*n* = 5).

Western blotting was used to quantify the abundance of Zo1, Occludin, and Claudin1 in the colonic tissues of mice to reflect the gut barrier function of mice. The abundance of Zo1, Occludin, and Claudin1 was maximum in the CD group and minimum in the RMD + EMS group, with the expression levels in the CD + EMS group and the RMD + EMS + SCFA group being intermediate (Figure 7A,B). These data indicate that a red meat diet can significantly disrupt gut barrier function in mice with endometriosis; however the intervention of SCFAs can reverse the damage to the intestinal barrier in mice with endometriosis caused by a red meat diet to a certain extent.

Recognizing the vital role of the gut barrier in preventing bacterial lipopolysaccharides (LPS) from entering systemic circulation and the peritoneal cavity is crucial (Camilleri and Vella 2022; Ni et al. 2021), therefore, we quantified the concentrations of LPS in both the serum and peritoneal cavity to accurately represent LPS concentrations within the circulatory and peritoneal environments.

**FIGURE 8:**
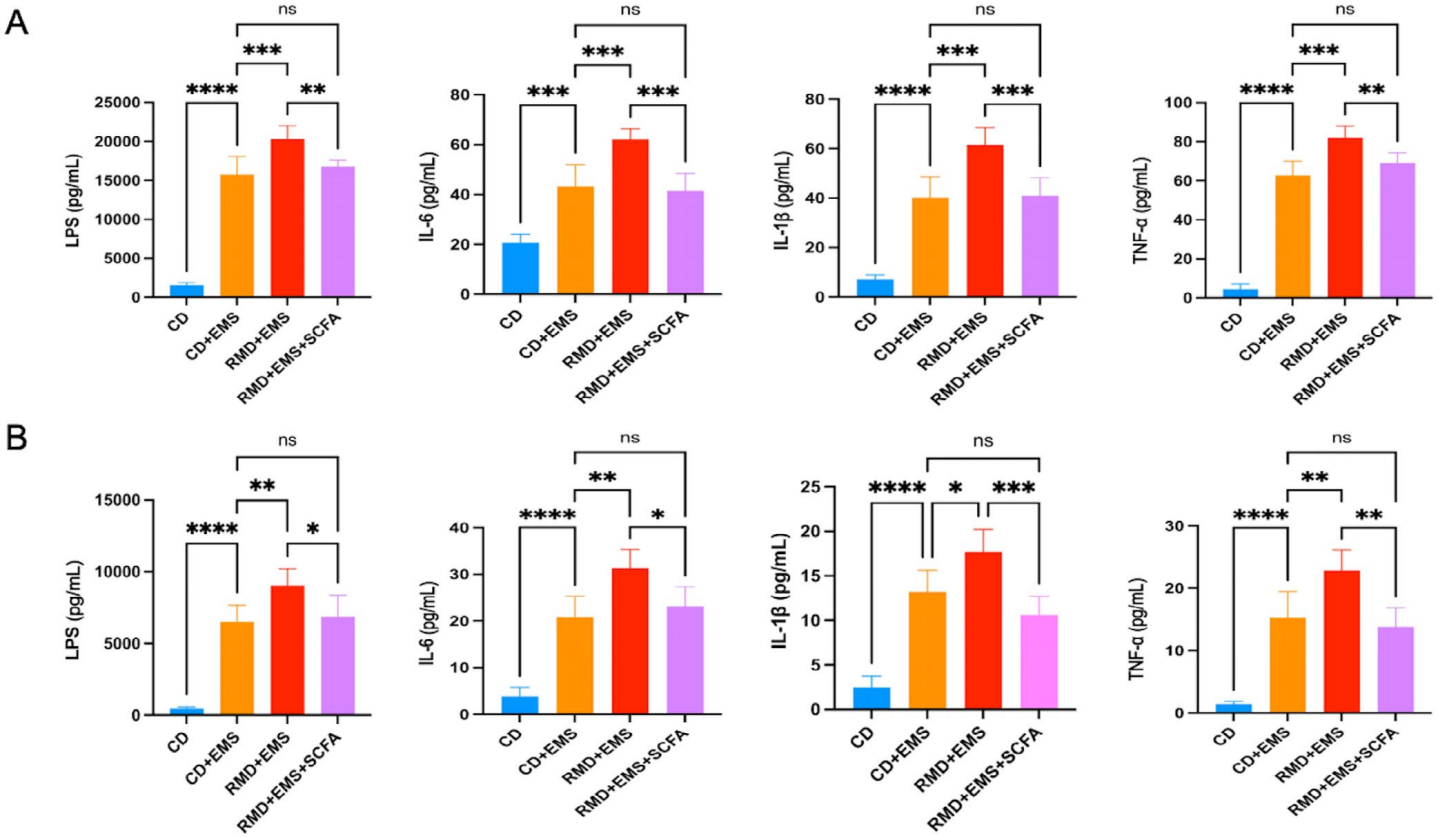
LPS, IL‐1β, IL‐6, and TNF‐α levels in the lavage fluid and serum of the CD, CD + EMS, RMD + EMS, and RMD + EMS + SCFA groups. (A) LPS, IL‐1β, IL‐6, and TNF‐α levels in the lavage fluid of each group. (B) Serum LPS, IL‐1β, IL‐6, and TNF‐α levels in each group. One‐way ANOVA. ns, non‐significant; *p < 0.05, **p < 0.01, ***p < 0.001, ****p < 0.0001 (*n* = 5).

Mice in the CD + EMS group showed an inflammatory state: compared with the CD group, the concentrations of LPS, IL‐1β, IL‐6, and TNF‐α in the serum and peritoneal cavity of mice in the CD + EMS group were substantially increased, and the intervention of a red meat diet further increased the levels of inflammatory factors (Figure 8). Nonetheless, administering SCFA led to a decrease in inflammatory markers in mice with endometriosis fed a red meat diet.

To investigate whether there was an elevation in inflammatory levels in the lesions of endometriosis mice, the expression levels of proteins correlated with the inflammation‐related pathway in the lesion tissues of endometriosis mice were detected using WB. Since the intestinal barrier of endometriosis mice was damaged and LPS levels in the circulation and peritoneal cavity were elevated, the abundance of proteins in the NF‐κB pathway was first detected. The abundance of TLR4, MYD88, and PP65/P65 was substantially increased in the RMD + EMS group compared to that in the CD + EMS group. Conversely, the expression of these proteins was notably lower in the RMD + EMS + SCFA group (Figure 7A,C). These findings indicate that a red meat‐rich diet may impair gut barrier function and worsen inflammation in mice with endometriosis.

### 3.5 The Effect of Microbiota After Red Meat Diet Intervention on Endometriosis in Mice

**FIGURE 9:**
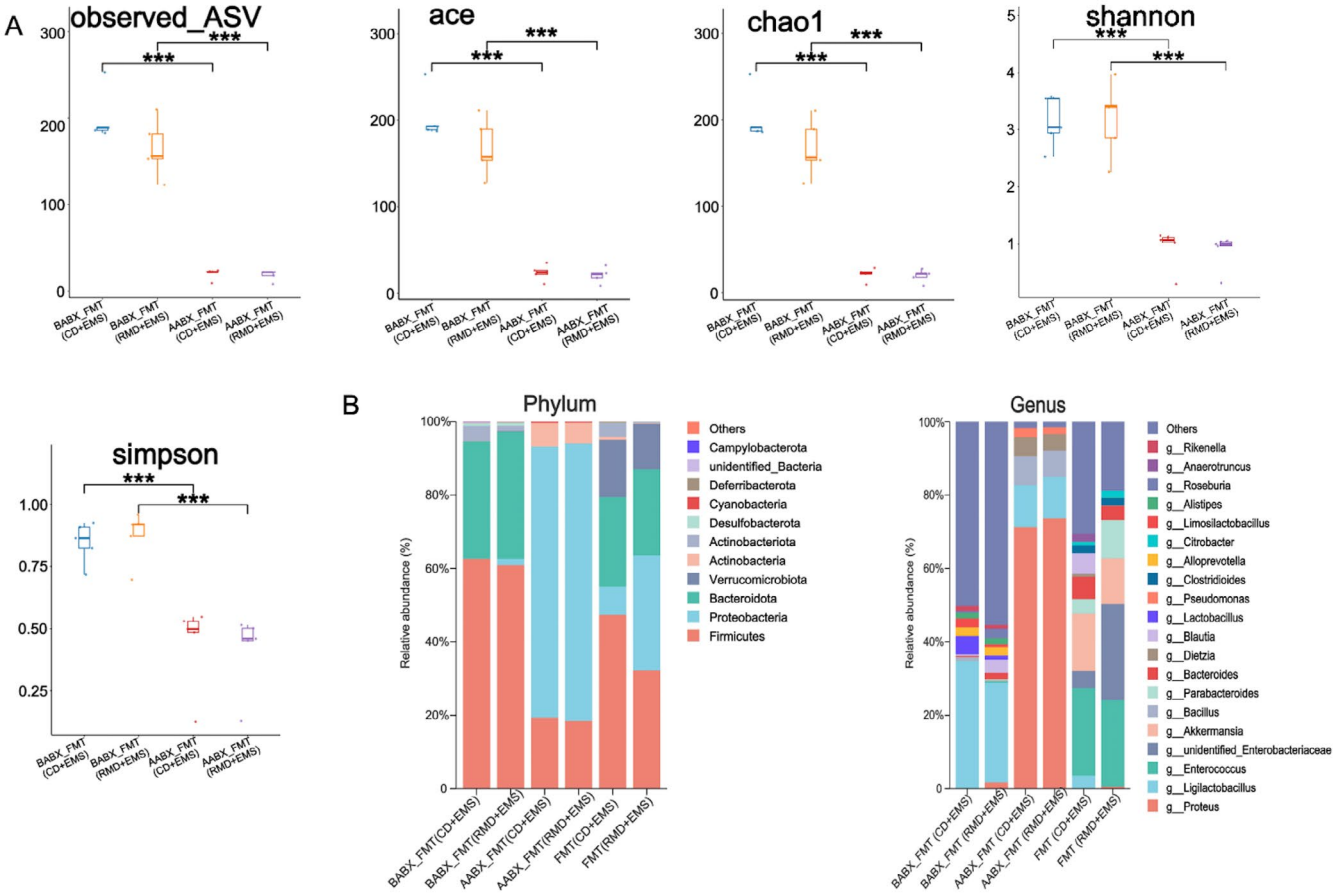
On Days 0, 14, and 61, the gut microflora of the FMT (CD + EMS) and FMT (RMD + EMS) groups. (A) Alpha diversity index of the gut flora in BABX_FMT (CD + EMS), AABX_FMT (CD + EMS), BABX_FMT (RMD + EMS), and AABX_FMT (RMD + EMS) groups. (B) Phylum‐ and genus‐level composition of the gut flora in each group. *p < 0.05, **p < 0.01, ***p < 0.001 (Kruskal–Wallis test) (*n* = 5). BABX_FMT (CD + EMS), the FMT (CD + EMS) group before antibiotic treatment; AABX_FMT (CD + EMS), the FMT (CD + EMS) group after antibiotic treatment; BABX_FMT (RMD + EMS), the FMT (RMD + EMS) group before antibiotic treatment; AABX_FMT (RMD + EMS), the FMT (RMD + EMS) group after antibiotic treatment (*n* = 5).

To assess whether alterations in intestinal microflora are causally linked to the progression of endometriosis, we conducted an FMT experiment, as detailed in the Methods and Materials section. Before and after the administration of antibiotics to the mice, and 3 weeks after fecal microbiota transplantation, we conducted 16S rRNA sequencing on fecal specimens from the two groups of mice. The number of ASVs decreased from 200 to 20 after the combined use of antibiotics (*p* < 0.01) in the FMT (CD + EMS) group (Figure 9A). Additionally, the species richness (Chao1 index) decreased from 201.762 to 21.317 (*p* < 0.01) (Figure 9A), and the diversity (Shannon index) decreased from 3.123 to 0.930 (*p* < 0.01) (Figure 9A). In the FMT (RMD + EMS) group, the number of ASVs decreased from 164.800 to 18.400 after the combined use of antibiotics (*p* < 0.01) (Figure 9A). Additionally, the species richness (Chao1 index) decreased from 167.224 to 19.600 (*p* < 0.01) (Figure 9A), and the diversity (Shannon index) decreased from 3.180 to 0.875 (*p* < 0.01) (Figure 9A), indicating that the antibiotic intervention depleted most of the resident intestinal flora in both groups of mice, successfully establishing a pseudo‐germfree mouse model.

**FIGURE 10:**
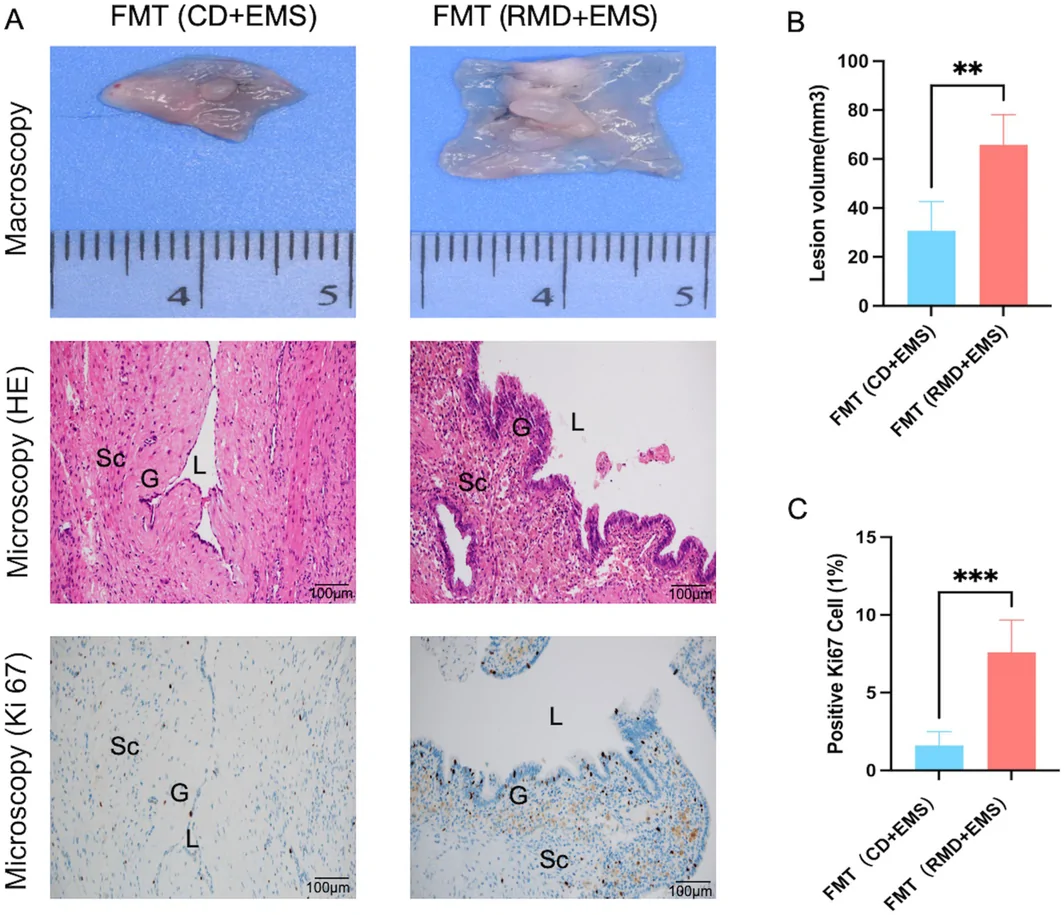
The promoting effect of intestinal flora in mice with endometriosis on endometriosis lesions. (A) Macroscopic observation images, HE staining images, and Ki67 immunohistochemical images of the implants in the FMT (CD + EMS) and FMT (RMD + EMS) groups on Day 61 (G, glandular cells; L, lumen; Sc, stromal cells). (B) Lesion volumes of the FMT (CD + EMS) and FMT (RMD + EMS) groups on Day 61. (C) Percentage of epithelial cells with positive Ki‐67 immunostaining in the FMT (CD + EMS) and FMT (RMD + EMS) groups. *t*‐Test. ns, non‐significant; *p < 0.05, **p < 0.01, ***p < 0.001. Statistics are shown as mean ± SE (*n* = 5).

We observed that the endometriotic lesions in the FMT (RMD + EMS) group were larger than those in the FMT (CD + EMS) group (Figure 10A,B). HE staining revealed that the mice in the endometriosis model construction group had typical endometriosis‐like structures. However, compared to the FMT (CD + EMS) group, the epithelial layer in the FMT (RMD + EMS) group was thicker, and the glands were more abundant (Figure 10A). Ki‐67 immunohistochemical staining was performed to assess cell proliferation (Figure 10A). The observations demonstrated that the proliferation index of the lesions in the FMT (RMD + EMS) group was substantially higher than that in the FMT (CD + EMS) group (*p* < 0.05) (Figure 10C).

Before starting the experiment, we analyzed the gut microbiome of each group of mice. Each group of mice was rich in Firmicutes and Bacteroidota at the phylum level (Figure 9B). Each group of mice was rich in *Ligilactobacillus*, *Lactobacillus*, and *Alloprevotella* at the genus level (Figure 9B). After 2 weeks of antibiotic use, at the phylum level, the most abundant species were Proteobacteria, Firmicutes, and Actinobacteria (Figure 9B). Each group of mice was rich in *Proteu*, *Ligilactobacillus*, *Bacillus*, *Dietzia*, and *Pseudomonas* at the genus level. (Figure 9B).

**FIGURE 11:**
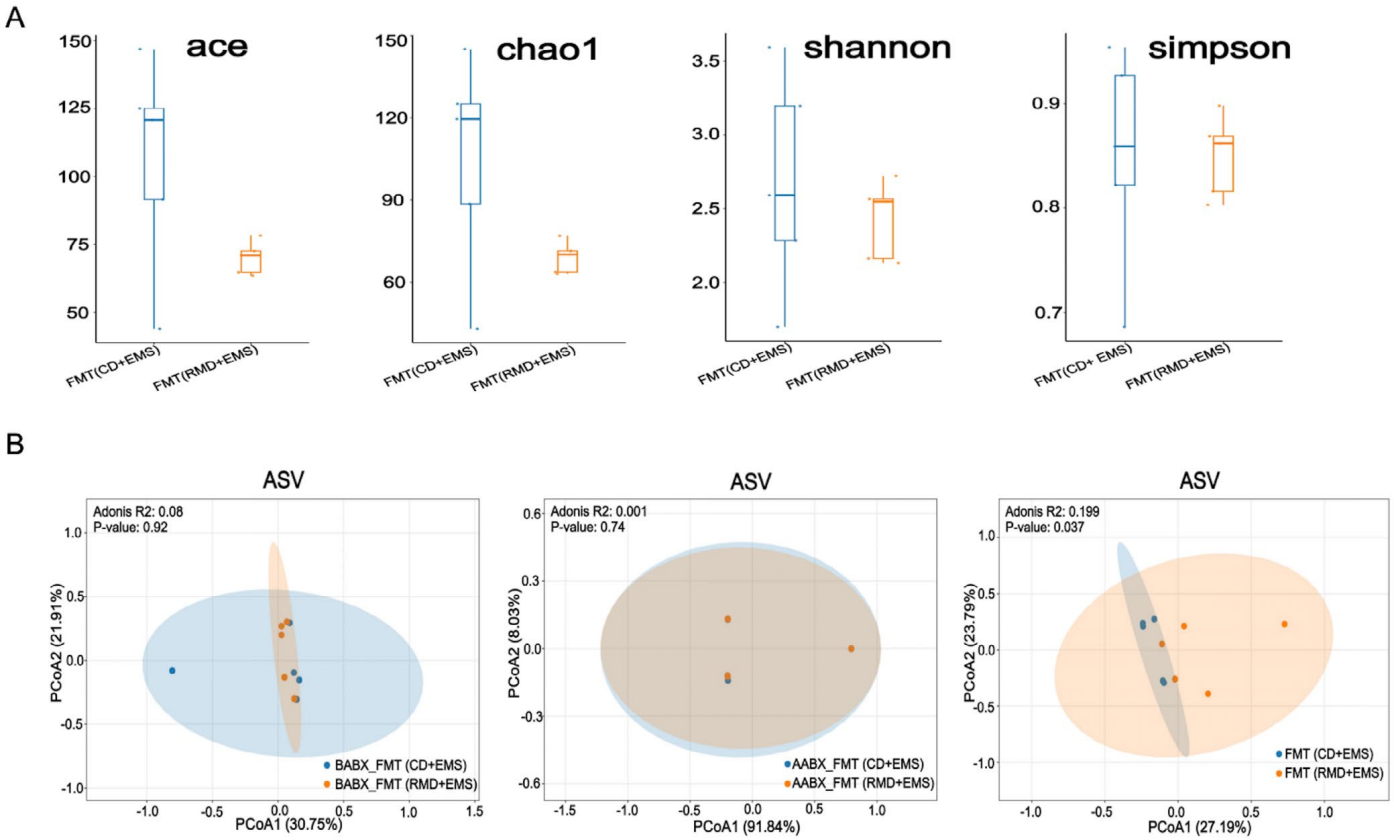
(A) On Day 61, the alpha diversity index of the gut flora in the FMT (CD + EMS) and FMT (RMD + EMS) groups. (B) On Days 0, 14, and 61, beta diversity of bacteria based on PCoA according to the Bray–Curtis distance between the two groups at the genus rank. *p < 0.05, **p < 0.01 (Kruskal–Wallis test or *t*‐test) (*n* = 5).

The PCoA of Bray–Curtis distance showed no clear distinction in the gut flora of the two groups of mice at the beginning of the trial (*R* = 0.08, *p* = 0.92) (Figure 11B), and after 2 weeks of antibiotic use, there was no meaningful separation in the gut flora of the two groups of mice (*R* = 0.001, *p* = 0.74) (Figure 11B). After concluding the experiment, we analyzed the gut flora of each group of mice. The FMT (RMD + EMS) group demonstrated a higher prevalence of Proteobacteria than the FMT (CD + EMS) group, whereas the Firmicutes and Actinobacteria showed lower abundances at the phylum level (Figure 9B). The intestinal flora composition of the two groups was also different at the genus level. The FMT (RMD + EMS) group exhibited an imbalance in the intestinal microbiota in contrast to the FMT (CD + EMS) group. This imbalance was marked by a decreased abundance of *Akkermansia*, *Bacteroides*, and *Ligilactobacillus*, whereas the levels of *unidentified_Enterobacteriaceae* and *Parabacteroides* were elevated (Figure 9B).

By calculating and comparing the alpha diversity indices of the gut flora of the two groups of FMT mice, the Ace, Chao1, Shannon, and Simpson indices indicated no statistical differences (Figure 11A), indicating no disparity in the species abundance and diversity of the gut flora in intergroup comparison.

PCoA of the Bray–Curtis distance demonstrated that the intestinal microbiota specimens of the FMT (RMD + EMS) and FMT (CD + EMS) groups were clearly divided into two clusters in the coordinate system (*R* = 0.199, *p* = 0.037) (Figure 11B), indicating that the general constitution of the gut flora in the two groups of mice was markedly different.

**FIGURE 12:**
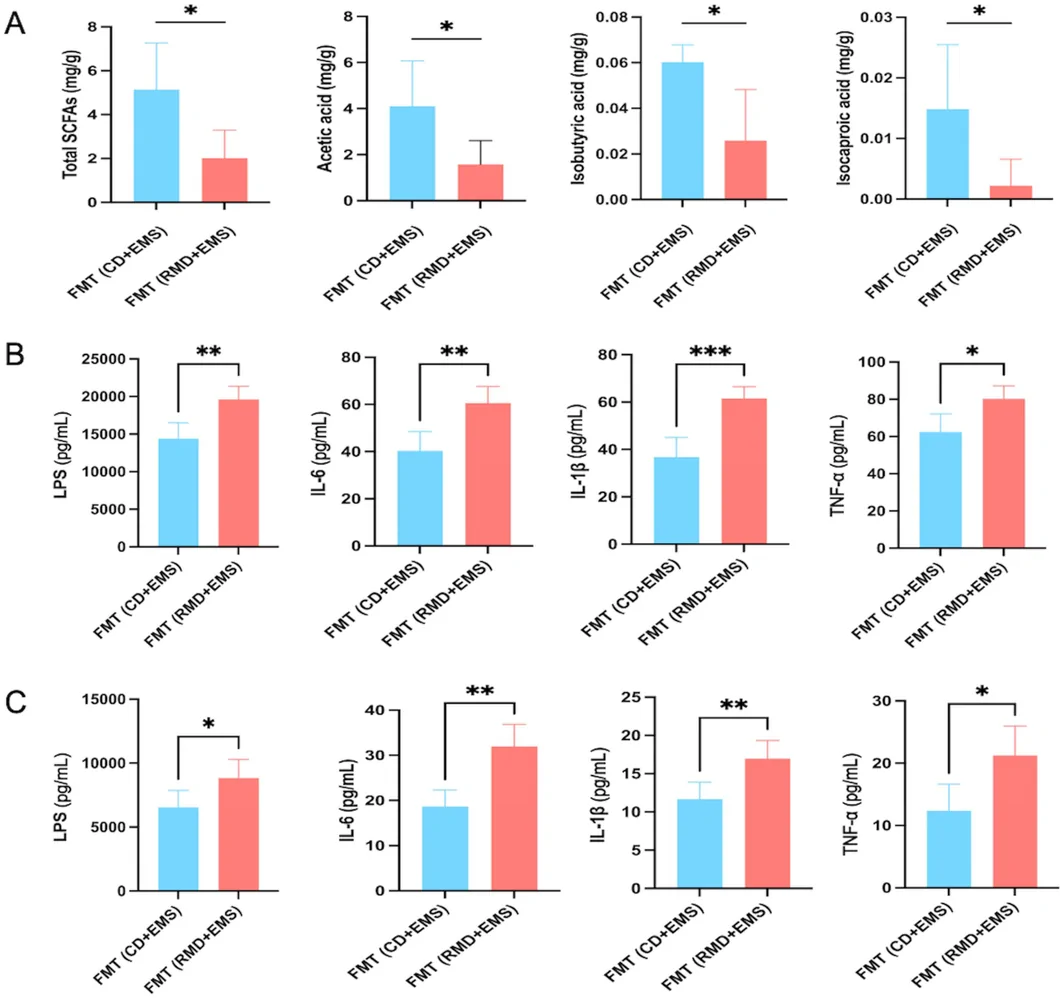
Lavage fluid and serum LPS, IL‐1β, IL‐6, and TNF‐α levels in the FMT (CD + EMS) and FMT (RMD + EMS) groups. (A) LPS, IL‐1β, IL‐6, and TNF‐α levels in the lavage fluid of each group. (B) Serum LPS, IL‐1β, IL‐6, and TNF‐α levels in each group. *t*‐Test. ns, non‐significant; *p < 0.05, **p < 0.01, ***p < 0.001.

**FIGURE 13:**
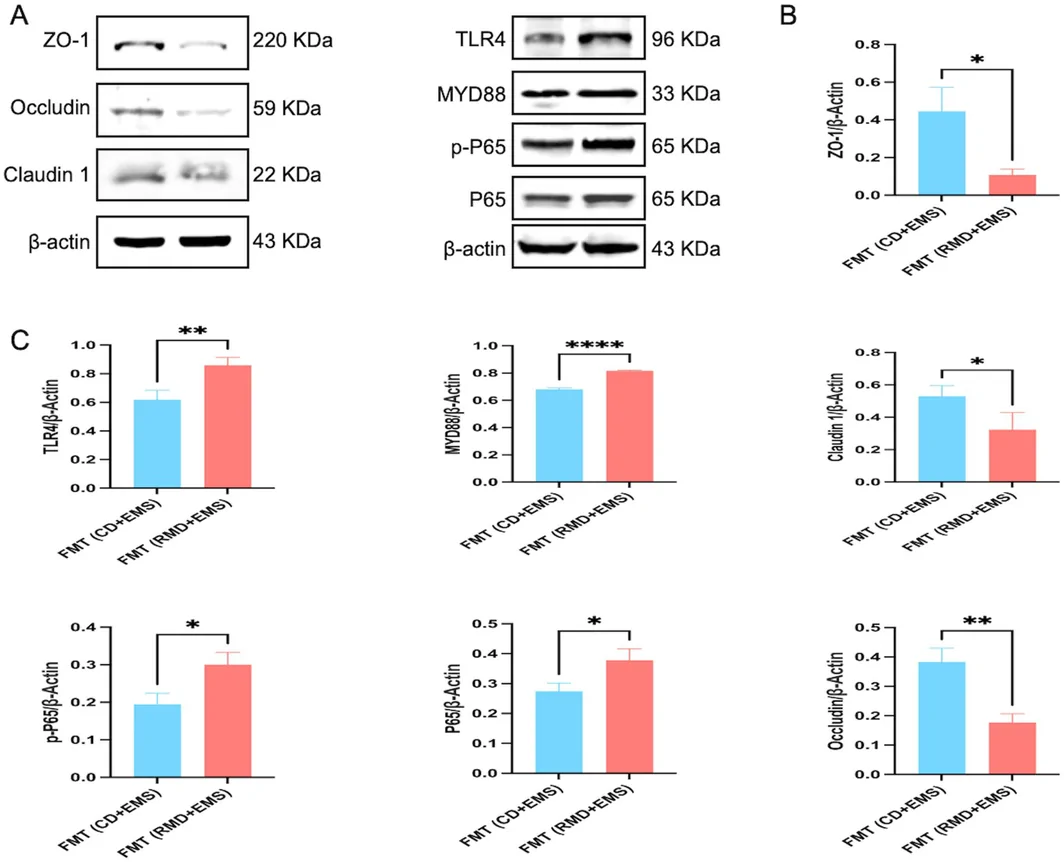
(A) Western blot analysis of intestinal barrier proteins (ZO‐1/Occludin/Claudin1) and the TLR4/MYD88/NF‐κB pathway in the FMT (CD + EMS) and FMT (RMD + EMS) groups. (B) Relative amounts of ZO‐1, Occludin, and Claudin1. (C) Relative amounts of TLR4, MYD88, p‐P65, and P65. *t*‐Test. ns, non‐significant; *p < 0.05, **p < 0.01, ***p < 0.001, ****p < 0.0001 (*n* = 5).

We also quantified the concentrations of SCFAs in fecal samples. The total SCFAs in the FMT (RMD + EMS) group of mice were significantly lower, including acetic, isobutyric, and isohexanoic acids, than those in the FMT (CD + EMS) group of mice (Figure 12A). The gut barrier function in the FMT (RMD + EMS) group of mice was notably compromised compared to that in the FMT (CD + EMS) group. This was evidenced by the decreased expression of genes responsible for encoding Zo1, Occludin, and Claudin1 proteins (Figure 13A,B). In terms of inflammation, the concentrations of pro‐inflammatory markers such as LPS, IL‐1β, IL‐6, and TNF‐α in the abdominal cavity and blood of the FMT (RMD + EMS) group of mice were notably elevated compared to those in the FMT (CD + EMS) group of mice (Figure 12B,C). WB evaluation of the abundance of inflammatory pathway‐related proteins in the lesion tissues of endometriosis mice in each group showed that in contrast with the FMT (CD + EMS) group of mice, the abundance of TLR4, MYD88, and PP65/P65 were remarkably elevated in the FMT (RMD + EMS) group (Figure 13A,C).

## 4 Discussion

This study established an endometriosis model using 8‐week‐old female C57 mice and evaluated the effects of a red meat diet on EMS progression by analyzing the lesion volume, intestinal microbiota composition, and inflammatory status. Our results demonstrated that RMD reshapes the gut microbiota, reduces short‐chain fatty acid production, impairs intestinal barrier integrity, and triggers systemic inflammation, thereby aggravating endometriosis development.

Epidemiological evidence has linked high red meat intake, a typical pro‐inflammatory dietary pattern, to an elevated EMS risk (Ley et al. 2014; Wu, Shinde, et al. 2021; Wu, Zhang, et al. 2021; Shiraseb et al. 2022; Samraj et al. 2015). A cohort of 81,908 participants reported a higher EMS risk in women who consumed ≤ 1 serving of red meat per week, while another large cohort confirmed that a Western diet rich in red meat significantly increased disease susceptibility (Yamamoto et al. 2018; Dougan et al. 2024). Moreover, rational dietary modulation can alleviate EMS‐related pain and improve patients' quality of life (Piecuch et al. 2022; van Haaps et al. 2023). Accordingly, dietary interventions are valuable for EMS prevention in high‐risk populations and as adjuvant clinical treatment.

A red meat diet is known to exacerbate diabetes and cardiovascular disorders by disturbing gut microbiota homeostasis (Fackelmann et al. 2025; Feng et al. 2015; Shi et al. 2023), whereas its mechanistic role in EMS remains poorly defined. In the present study, EMS modeling alone induced gut dysbiosis, with decreased abundance of Ligilactobacillus and increased abundance of conditional pathogens, including *Enterococcus* and Enterobacteriaceae, consistent with previous reports (Bailey and Coe 2002; Leonardi et al. 2020; Hu et al. 2023). RMD further deteriorated this microbial imbalance, and SCFA supplementation partially reversed the overgrowth of Enterobacteriaceae. This observation is supported by a clinical trial showing that dietary addition of butyrate‐producing starch reduced intestinal Enterobacteriaceae abundance in populations consuming a high red meat diet (Le Leu et al. 2015).

As a key probiotic genus, Ligilactobacillus is critical for maintaining gut homeostasis and host health (Chuandong et al. 2024). The loss of Lactobacillus dominance is a typical feature of EMS‐related dysbiosis (Jiang et al. 2021). Conversely, Enterobacteriaceae is closely implicated in multiple inflammatory diseases (Baldelli et al. 2021; Xu et al. 2021; Shen et al. 2017). Clinically, patients with EMS frequently present with intestinal dysfunction, with significantly higher intestinal and peritoneal *Enterococcus* abundance than healthy individuals (Seaman et al. 2008; Hu et al. 2023; Zhu et al. 2024). Relevant therapy can remodel the microbiota toward an anti‐inflammatory and barrier‐protective phenotype (Pronina et al. 2025).

Acetic acid was the dominant fecal SCFAs in our study. Correlation analysis showed that Blautia, Ligilactobacillus, Alloprevotella, and Alistipes were positively correlated with acetic acid levels, while Enterobacteriaceae and *Enterococcus* were negatively associated with SCFA contents, which is in accordance with prior studies (Ye et al. 2023; Guo et al. 2025; Wu, Shinde, et al. 2021; Wu, Zhang, et al. 2021; Yin et al. 2018; Garrett et al. 2010; Buttler et al. 2025). Although Akkermansia is recognized as a classic SCFA‐producing bacterium, no significant positive correlation was observed between its abundance and acetic, propionic, or butyric acid in this study. This may result from substrate competition with high‐efficiency SCFA‐producing bacteria or the enrichment of SCFA‐consuming taxa in Akkermansia‐dominated communities, reflecting the complexity of internal microbial interactions.

SCFAs, which are critical fermentative metabolites of dietary fiber, maintain gut barrier integrity and regulate systemic immune and inflammatory responses (Mann et al. 2024; Kim 2023). Gut dysbiosis is tightly associated with increased intestinal permeability and abnormal inflammation (Zheng et al. 2020; Belkaid and Hand 2014; Zhao et al. 2023; Martel et al. 2022). In EMS mice, RMD inhibited the expression of tight junction proteins ZO‐1, Occludin, and Claudin‐1, damaging the intestinal barrier structure, whereas SCFA supplementation effectively alleviated such injury. Barrier disruption allows leakage of LPS and bacterial components into the circulation and peritoneal cavity, triggering systemic inflammation. Consistently, we detected elevated levels of LPS and pro‐inflammatory cytokines IL‐1β, IL‐6, and TNF‐α in EMS mice, confirming the inflammatory nature of endometriosis.

Chronic inflammation is the core driver of EMS occurrence and progression, with increased pro‐inflammatory cytokines widely detected in patient circulation, peritoneal fluid, and ectopic lesions (Dai et al. 2025; Blanco et al. 2025; Wang, Nicholes, and Shih 2020; Wang, Zhang, et al. 2020; Martínez et al. 2007; Kang et al. 2014; Malvezzi et al. 2022; Keenan et al. 1995; Bedaiwy et al. 2002). Mechanistically, LPS activates the TLR4/MyD88/NF‐κB pathway, in which p65 phosphorylation serves as a key activation marker (Wang, Nicholes, and Shih 2020; Wang, Zhang, et al. 2020). This pathway is involved in EMS pathogenesis and pain modulation (Liu et al. 2022; Su et al. 2021). Our findings validated NF‐κB pathway activation in EMS lesions; RMD further enhanced this activation, whereas SCFA intervention exerted a significant inhibitory effect.

Fecal microbiota transplantation was performed to clarify the causal relationship between the RMD‐altered microbiota and EMS. Even under a normal diet, mice receiving microbiota from RMD‐treated EMS donors displayed aggravated lesions, histological damage, gut barrier dysfunction, and systemic inflammation compared with the control FMT group. These findings confirm that RMD reshaped the gut microbiota through RMD and is an independent causal factor promoting EMS progression. FMT recipients with RMD‐related dysbiosis showed reduced abundance of Akkermansia, Bacteroides, and Lactobacillus and increased abundance of Enterobacteriaceae and Parabacteroides. The decline in Bacteroides abundance is consistent with the microbial characteristics of patients with advanced EMS (Cai et al. 2025). Collectively, our results construct a clear mechanistic chain: microbiota dysbiosis, SCFA reduction, intestinal barrier destruction, inflammatory activation, and EMS aggravation.

To our knowledge, this is the first study to explore the effect and mechanism of RMD on EMS by regulating the gut microbiota. We demonstrated that RMD facilitates the proliferation of pathogenic bacteria, suppresses SCFA‐producing taxa, reduces intestinal SCFA content, disrupts gut barrier homeostasis, and activates inflammatory responses, thereby accelerating EMS progression. This study provides experimental evidence for dietary regulation and microecological intervention as potential auxiliary strategies for EMS prevention and treatment and offers a new perspective for understanding the interplay among diet, gut microbiota, and chronic gynecological inflammation.

This study had several limitations. First, all data were obtained from mouse models, and interspecies physiological differences restrict direct clinical extrapolation; further verification in human cohorts is required to confirm these findings. Second, we focused mainly on the TLR4/NF‐κB pathway, while other inflammatory signaling pathways may also be involved. Third, the short experimental period failed to evaluate the long‐term effects of RMD on EMS recurrence and fertility. Fourth, the absence of an independent RMD‐only control group prevented the distinction of the separate effects of RMD and EMS on the microbiota and inflammation. Fifth, the relatively small sample size may limit the statistical reliability and generalizability of the findings. Future large‐sample studies and clinical validation are required to complement and confirm our conclusions.

## 5 Conclusions

In summary, our study is the first to establish a complete mechanism chain of “red meat diet‐dysbiosis‐reduction of SCFA‐barrier damage‐activation of inflammation‐aggravation of the disease,” providing an important theoretical basis for dietary intervention in endometriosis.

## Author Contributions

**Qian Peng:** formal analysis, investigation, visualization. **Wenwei Chen:** validation. **Suting Li:** funding acquisition, resources. **Qian Liu:** methodology, supervision. **Jinhua Tian:** formal analysis. **Mengru Li:** conceptualization, formal analysis, visualization, writing – original draft, data curation. **Yang Li:** methodology, project administration. **Jinxin Xiao:** software. **Aili Tan:** resources. **Qingzhen Xie:** funding acquisition, project administration, writing – review and editing.

## Funding

The National Natural Science Foundation of China (nos 82401896 and 210972408) provided financial support for this work.

## Conflicts of Interest

The authors declare no conflicts of interest.

## Data Availability

The datasets used and/or analyzed during the current study are available from the corresponding author on reasonable request.
